# Advances in pediatrics in 2014: current practices and challenges in allergy, gastroenterology, infectious diseases, neonatology, nutrition, oncology and respiratory tract illnesses

**DOI:** 10.1186/s13052-015-0193-8

**Published:** 2015-10-31

**Authors:** Carlo Caffarelli, Francesca Santamaria, Silvia Cesari, Elisa Sciorio, Carlotta Povesi-Dascola, Sergio Bernasconi

**Affiliations:** Clinica Pediatrica, Department of Clinical and Experimental Medicine, University of Parma, Parma, Italy; Department of Translational Medical Sciences, Federico II University, Naples, Italy; Pediatrics Honorary Member University Faculty, “G D’ Annunzio” University of Chieti Pescara, Chieti, Italy

**Keywords:** Allergy, Gastroenterology, Infectious diseases, Neonatology, Nutrition, Oncology, Respiratory tract illnesses

## Abstract

Major advances in the conduct of pediatric practice have been reported in the Italian Journal of Pediatrics in 2014. This review highlights developments in allergy, gastroenterology, infectious diseases, neonatology, nutrition, oncology and respiratory tract illnesses. Investigations endorse a need to better educate guardians and improve nutritional management in food allergy. Management of hyperbilirubinemia in neonates and of bronchiolitis have been improved by position statements of scientific societies. Novel treatments for infant colic and inflammatory bowel diseases have emerged. Studies suggest the diagnostic utility of ultrasonography in diagnosing community-acquired pneumonia. Progress in infectious diseases should include the universal varicella vaccination of children. Recommendations on asphyxia and respiratory distress syndrome have been highlighted in neonatology. Studies have evidenced that malnutrition remains a common underestimated problem in developing countries, while exposure to cancer risk factors in children is not negligible in Western countries. Advances in our understanding of less common diseases such as cystic fibrosis, plastic bronchitis, idiopathic pulmonary hemosiderosis facilitate diagnosis and management. Researches have led to new therapeutic approaches in patent ductus arteriosus and pediatric malignancies.

## Background

This review focuses on developments in allergology, gastroenterology, infectious diseases, neonatology, nutrition, oncology and respiratory tract illnesses that will influence the practice of pediatrics. Studies were identified among the most-accessed articles published in 2014 in the Italian Journal of Pediatrics.

## Review

### Allergy

Although food allergy is common in childhood, its nutritional management has been given limited prominence in pediatric research [1]. However, in population-based studies, growth deficiency and malnutrition have been shown to be common in children with cow’s milk allergy, wheat allergy or multiple food allergies [2]. Giovannini et al. [3] recommend regular visits for updating on nutritional requirements and monitoring growth. They suggest nutritional interventions and pharmacological supplementation when dietary modifications are inadequate to meet vitamin, mineral, and trace element needs. Supplementation with calcium and vitamin D are generally required in children with cow’s milk allergy [2]. Giovannini et al. [3] suggest that a multidisciplinary approach, including the interaction of nutritionists, dieticians, nurses, allergists and psychologists, is necessary to ensure growth in food allergic children and help family members deal with the exclusion of offending foods. Furthermore, future challenges include the evaluation and confirmation of dietetic regimens associated with better clinical outcomes, and the development of methods for identifying diet-responsive and nonresponsive phenotypes.

### Gastroenterology

Even though infantile colic is a benign transient condition, more attention should be given to the high burden of the affection. A complete understanding of the pathogenesis is however lacking. Thus, no consensus has been reached on its management and treatment. Contrasting data have been reported on the usefulness of maternal milk-free diet or the use of probiotics on the decrease in daily crying episodes in these infants [4, 5]. Savino et al. [6] propose different dietary approaches based on the type of feeding. Breast-feeding mothers should go on a milk-free diet for 2 weeks. If the diet does not work, probiotics and pain relieving agents such as simethicone and cimetropium bromide should be given. In formula-fed infants, a partially hydrolyzed formula with prebiotics or probiotics is suggested. If the treatment is unsuccessful, an extensively hydrolyzed formula should be administered. Evidence supporting the use of complementary and alternative treatments (herbal supplements, manipulative approach and acupuncture) or behavioral interventions is limited. The authors conclude that further studies are required in order to improve knowledge on infantile colic treatment.

Dysbiosis is characteristic of inflammatory bowel disease (IBD) and the largest genetic IBD study suggests that genetic response to the gut microbiome plays a pathogenetic role [7]. The microbiota of the gut has an anti-inflammatory effect on the developing immune system [8]. Comito et al. [9] underline that it is also involved in digestion of dietary components, vitamin synthesis and intestinal motility. An abnormal colonization of the ileal mucosa is associated with severity and risk of reactivation of ileal Crohn’s disease [9]. Microbial imbalance causes low intraluminal levels of butyrate, down-regulation of epithelial tight junction protein expression and increased epithelial permeability. This leads to impairment of bacteria killing with excessive Toll-like receptor stimulation, secretion of pro-inflammatory cytokines and activation of innate and T-cell mediated immune responses with induction of intestinal inflammation by commensal bacteria and pathogens [9]. Several genes, such as NOD2, IL-23 R and ATG16L1 are involved in loss of processing products of resident intestinal microbiota which may lead to lasting immune response. Overall, dysbiosis may contribute to the development of IBD but may also be a result of the disease. Antibiotic therapy has not been demonstrated to be effective in the treatment of IBD, except in specific circumstances. Therefore, changing bacterial populations by probiotics or fecal microbiota transplantation seem to be promising treatments in IBD that deserve further investigations [9].

### Infectious diseases

The most common neurological complication of varicella is acute cerebellitis, developing in 1:4000 varicella cases [10]. The diagnosis of acute cerebellitis is based on the occurrence of ataxia following a chickenpox rash within 3 weeks of varicella onset. Bozzola et al. [11] report that 48 (10.5 %) out of 457 children hospitalized for varicella in a 10 year time interval presented acute cerebellitis. This rate is in agreement with previous studies. They described frequency of symptoms. In the 48 cases, symptoms were as follows: ataxia (95 %), slurred speech (37.5 %), vomiting (31.25 %), headache (29.16 %), dysmetria (25 %) and tremor (22.91 %). Nystagmus or other involuntary eye movements rarely occurred. Even though laboratory investigations are not considered necessary for diagnosis, three patients underwent a lumbar puncture, 18 magnetic resonance imaging and two computed tomography. Such investigations, however, did not show relevant findings. In addition to intravenous acyclovir, 33 % of subjects received systemic steroids that did not improve symptoms. At the 2-month follow-up, one child showed persistent unintentional tremors. No child in the study population had received the varicella vaccine. This reflects low vaccination coverage in the region from which the patients included in the study came from. These data concur to demonstrate that universal varicella vaccination of children is beneficial not only in the perspective of preventing varicella complications but also in reducing societal costs [12].

Community-acquired pneumonia (CAP) is the most common disease recorded worldwide. The diagnosis is established, in an appropriate clinical setting, by the finding of a new pulmonary infiltrate on chest radiograph. Lung ultrasound represents a new technique used for diagnosing pleural and pulmonary diseases [13]. It is a noninvasive tool, and is usually available in most hospitals. This is especially important if radiography is not available or applicable, and only 8 % of pneumonic lesions are not detected by lung ultrasound [14]. Presently only few prospective studies on point-of-care ultrasonography (US) in pediatric CAP have been published. In particular, very few data on the efficacy of US compared to conventional chest radiography (CR) in defining different types of lung abnormalities have been reported. According to recent guidelines, the diagnosis of mild to moderate cases of pediatric CAP can be based solely on clinical criteria, whereas CR is required in severe cases which need hospitalization and/or when complications are suspected. The absence of CR confirmation leads to an overestimation of the incidence of CAP causing a number of problems, the most important of which being an increased use of unnecessary antibiotics. In an attempt to overcome this problem, US was proposed in 1986, but only recently technological advances revived the interest in US as a diagnostic tool for lower respiratory tract infections. A number of studies have shown that it is feasible and accurate if used by experienced clinician-sonologists, but there are still few prospective evaluations of point-of-care US for the diagnosis of pediatric CAP. Esposito et al. [15] prospectively studied 103 children who were admitted to the hospital with a clinical suspicion of CAP. Patients underwent CR (evaluated by an independent expert radiologist) and lung US (performed by a resident in pediatrics with limited experience in US). The accuracy of US in diagnosing CAP (i.e. its sensitivity, specificity, and positive and negative predictive values) was compared with that of CR. Forty-eight of the 103 enrolled children had radiographically confirmed CAP. The findings of this study confirm that lung US is a simple and reliable technique that is nearly as reliable as CR in identifying lung changes that are diagnostic for CAP, and shows that it is even more effective than CR in diagnosing pleural effusion. These data further support recent recommendations of the International Liaison Committee on Lung Ultrasound on the use of US in pediatric patients with suspected CAP in order to reduce antibiotic abuse. US has a number of advantages: it is easy to perform at a child’s bedside, takes little time to perform and interpret results, allows a close follow-up and avoids the use of ionising radiation, which is particularly important in children who are at least four times more sensitive than adults to the induction of cancer. The concordance of the US and CR data was relatively limited in terms of type of lung lesion revealed, and this may have important implications when an antibiotic prescription is indicated. US may be considered as a useful means of diagnosing CAP in children admitted to an Emergency Department with lower respiratory tract infections. However, its usefulness in identifying the type of lung involvement is uncertain and may be influenced by the operator’s learning curve. Further evaluations are necessary before US is recommended in the decision making process concerning treatment of pediatric CAP.

*Mycoplasma pneumoniae* (MP) causes up to 40 % of community-acquired pneumonia (CAP) in children and about 18 % of infections in patients who require hospitalization. Compared with non-mycoplasma infections, CAP patients infected with MP are younger, less likely to have comorbidities and present a longer duration of fever before admission. In the majority of cases of suspected MP pneumonia, the presumptive diagnosis is performed on clinical and radiological findings alone. The study of Medjo et al. [16] included 166 children aged between 1 and 15 years with radiologically confirmed pneumonia. Authors showed that there were no characteristic radiological findings or routine laboratory tests that distinguished CAP caused by MP from other organisms. MP infection was more present in boys than in girls (*p* = 0.014). Children with MP pneumonia were significantly older (*p* = 0.001) and had longer duration of fever (*p* = 0.021) and cough before enrolment (*p* = 0.026) compared to those with non-MP pneumonia. Cough (*p* = 0.029), headache (*p* = 0.001) and wheezing (*p* = 0.036) were more frequent in children with MP pneumonia compared to other cases. Detection of IgM antibodies combined with real-time PCR allows a precise and reliable diagnosis of MP infection in children during the early acute phases of the disease, indicating a possible use of both techniques as a valid diagnostic approach. A recent study found a good agreement between positive rate of MP cultivation of throat swabs and acute MP infection, and concluded that the logical approach would be to incorporate PCR, culture and serological tests for optimum diagnosis of acute MP infections in adults and adolescents [17]. A systematic review showed that the presence of chest pain more than doubles the probability of MP pneumonia [18]. However, further research is needed to substantiate this finding. More high quality large-scale studies in primary care settings are needed to help develop prediction rules based on epidemiological data as well as clinical and baseline patient characteristics.

### Neonatology

Birth asphyxia represents a serious problem worldwide and contributes greatly to neonatal mortality and morbidity. According to the WHO, 4 million deaths that occur yearly are due to birth asphyxia, representing 38 % of all deaths in children <5 years of age. Among different countries, prevalence of birth asphyxia greatly varies in relation with socio-economical level [19]. Birth asphyxia is strongly associated with intrapartum stillbirth and is responsible for long-term neurological disability and impairment. Asiam et al. [20] conducted a retrospective case control study from January 2011 to November 2012 in the neonatal intensive care unit of the Civil Hospital of Karachi, Pakistan which aimed to evaluate antepartum, intrapartum, and fetal risk factors of birth asphyxia. The study evidenced maternal age, pre-eclampsia, and diuretic or adrenergic drug use as maternal risk factors. Intrapartum risk factors included home delivery by midwives, breech presentation, prolapsed umbilical cord, cephalopelvic disproportion and maternal fever. Prematurity, low birth weight, oligohydromnios, and meconium stained amniotic fluid were evidenced as fetal risk factors. This study emphasizes that the majority of these risk factors may be manageable by means of good pre-natal care. There is a need of interventions that focus on educating mothers during the antenatal period to reduce potential risk factors for mortality, especially in resource-limited settings [21].

A persistently patent ductus arteriosus (PDA) has significant clinical consequences in preterm neonates during the recovery period from respiratory distress syndrome. Ductal patency is regulated by circulating prostaglandins produced by prostaglandin-H2 synthetase, an enzyme which is composed of two active sites: cyclo-oxygenase (COX) and peroxidase. Indomethacin and ibuprofen are COX inhibitor drugs that are commonly used in the treatment of hemodynamically significant PDA. These drugs are however often associated in early life with serious adverse events, including gastrointestinal perforation, renal failure and bleeding. There is increasing interest in the role of paracetamol, an inhibitor of the peroxidase component of prostaglandin-H2 synthetase, for the treatment of PDA in the last few years following the first published case report in 2011 [22]. Terrin et al. [23] aimed to evaluate the efficacy of paracetamol in the early treatment of PDA in preterm neonates presenting contraindications to COX-inhibitors. In their series of eight neonates successful closure of the ductus arteriosus was achieved in six out of 8 babies (75 %) treated with paracetamol. On the basis of these results, the Authors state that paracetamol may be considered as a promising and safe choice in the treatment of PDA in neonates. Their findings are in agreement with results of controlled trials demonstrating that paracetamol and ibuprofen have similar efficacy in PDA treatment [24, 25].

Kangaroo mother care (KMC) is a method of holding a neonate in skin-to-skin contact, prone upright on the maternal chest. This method promotes health effects for both the mother and baby, including a decrease in morbidity and mortality, increase in breastfeeding, weight gain and mother-baby bonding. To date, most studies on KMC have addressed cardiorespiratory parameters rather than cerebral hemodynamics. Afaf et al. [26] evaluated the changes of cerebral blood flow in the middle cerebral artery, before and after a 30 min application of KMC in stable preterm infants. They evidenced an improvement in cerebral blood flow, within its normal level, with a significant decrease in both Pulsatility index and Resistive index after 30 min of KMC. This suggests a positive influence on the structure and development of the premature infant’s brain. Clinical evidence shows the usefulness of KMC in low birth weight infants as an alternative to conventional neonatal care mainly in developing countries [27]. However, KMC is physically and emotionally difficult, thus its practice needs to be supported by family members and health practitioners [28].

### Nutrition

Malnutrition is an unbearable burden not only on health systems, but on the entire socio-cultural and economic status of society, constituting a major public health problem in developing countries and contributing significantly to morbidity and mortality especially among preschool children. Globally, it is estimated that nearly 20 million children are severely malnourished, most of them living in south Asia and in sub-Saharan Africa. Worldwide malnutrition estimation rates indicate that 35.8 % of preschool children in developing countries are underweight, 42.7 % are stunted, and 9.2 % are wasted [29].

Manyike et al. [30], in a cross-sectional study, aimed to assess the prevalence of malnutrition and associated factors among children aged 1–5 years attending nursery and primary schools in Abakiliki in Ebonyi state of Nigeria. Anthropometry and clinical examination was performed and malnutrition was defined as a state of nutrition where weight for age, height for age and weight for height indices were below −2 Z-score of the NCHS references. The prevalence of global and severe acute malnutrition using z-score in the study was 9.7 and 4.4 % respectively while that of stunting was 9.9 % with a male preponderance. Reduced survival, increased incidence of acute and chronic diseases and impaired healthy development caused by malnutrition should lead to interventions that focus on effective nutrition programs [31].

### Oncology

The incidence of all cancers occurring in children under 20 years of age is about 175 cases per million in the United States, with an incidence of many individual types in the low dozens [32]. Rarity is thus a central factor which dictates the quality and quantity of evidence for causal associations between putative risk factors and childhood cancers. Most etiologic investigations on childhood cancer have therefore used the case–control study design, in which the characteristics of patients with a disease are compared to those of a carefully selected group of disease-free controls.

Given the milieu for childhood cancer epidemiology, evidence regarding causal associations has accumulated slowly, and etiology of childhood leukaemia and childhood neoplasm is still poorly understood. Information on the prevalence of risk factors in the pediatric population is limited as well. A multicenter, population-based case–control epidemiological study (SETIL - Studio Epidemiological Study on lymphoemopoietic cancers in childhood) was carried out to investigate risk factors for selected childhood neoplasms in Italy [33]. The primary study focused on leukaemia and was accompanied by two smaller studies on NHL and neuroblastoma. The study relied on questionnaire interviews and 50 Hz magnetic field (ELF-MF) indoor measurements. SETIL was carried out in 14 Italian regions (Piedmont, Liguria, Lombardy, The Venetian Region, Friuli Venezia Giulia, Emilia Romagna, Tuscany, Umbria, The Marches, The Latium, Campania, Apulia, Sicily and Sardinia). Cases of leukaemia, non-Hodgkin Lymphoma (NHL) and neuroblastoma in children aged 0–10 years and diagnosed between August 1998 and December 2001 (with minor regional differences) were eligible for recruitment. Two controls for each leukaemia case were randomly sampled from the Local Health Authority records, matched by gender, birthdate and residence. The same controls were used in NHL and neuroblastoma studies. Parents were interviewed at home on: physical agents (ELF-MF and ionizing radiation), chemicals (smoking, solvents, traffic, insecticides), occupation, medical and personal history of children and parents, infectious diseases, immunizations and associated factors. Occupational exposure was collected using job specific modules. ELF-MF was measured in the main rooms (spot measurement) and close to child’s bed (48 h measurements). The results showed that in Italian children the exposure to cancer risk factors is not negligible, and this appears to be consistent with data from other countries of Western lifestyle and industrial economy. Few environmental risk factors for childhood cancer have been identified in which observational epidemiology is able to distinguish between causal associations and associations due to bias [34]. Therefore, the association of common risk factors such as smoking with childhood leukemia is debated, even if they clearly represent risk factors for other diseases in childhood, such as asthma and acute respiratory illness.

### Respiratory tract illnesses

Viral bronchiolitis is the most frequent lower respiratory tract infection in infants and Respiratory Syncytial Virus (RSV) is the most common infecting agent, but other respiratory viruses may occasionally cause the disease as co-infecting agents. Bronchiolitis is the leading cause of hospitalization among infants during the first 12 months of life (with a peak of hospitalization at the age of 2 months), and a number of these children require admission to intensive care units and mechanical ventilation. Severe forms of the disease requiring hospitalization may be more frequent in children younger than 3 months of age or in children with pre-existing risk factors such as prematurity, bronchopulmonary dysplasia, congenital heart disease and immunodeficiency. In October 2006, the American Academy of Pediatrics published the clinical practice guideline “Diagnosis and Management of Bronchiolitis” [35]. The guideline offered recommendations ranked according to level of evidence and benefit-harm relationship. Since the completion of the original evidence review in July 2004, a significant body of literature on bronchiolitis has been published. However, there is no evidence of efficacy for numerous therapies commonly used when treating bronchiolitis (bronchodilators, steroids, antibiotics), and supportive treatment (oxygen and hydration) still remains the recommended approach, as confirmed by the very recent leading international guideline [36].

An intersociety consensus document [37] from Italy provides a multidisciplinary update for the National Health System (NHS), pediatricians working in hospital and emergency departments, primary care pediatricians, physicians attending postgraduate schools, nurses and all healthcare providers in the pediatric area, concerning scientific evidence on treatment and prevention of bronchiolitis, with a special focus on high risk pediatric populations. Authors will update the document every 3 years. Baraldi et al. [37] point out that milder forms of bronchiolitis may be adequately managed in the outpatient setting by primary care pediatricians, thus limiting hospital admissions. In the outpatient setting the child’s general clinical conditions must be assessed, together with his/her ability to feed, heart rate, respiratory rate, oxygen saturation (measured by pulse oximetry with specific sensors for infants), presence of risk factors and family compliance. Hospitalization is warranted based on the following conditions: O_2_ saturation persistently lower than 90–92 %, entity of respiratory distress and presence of apnea. Other important factors to take into consideration are: prematurity, gestational age <37 weeks or birth age <6–12 weeks, responsivity and alertness, decreased hydration and feeding, and preexisting chronic disorders. Children with severe bronchiolitis must be transferred to the Pediatric Intensive Care Unit, based on the following conditions: respiratory failure requiring mechanical ventilation, apnea with desaturation, severe impairment of general conditions. Criteria that must be taken into account for patients’ discharge include: sustained autonomy from any kind of respiratory support and O_2_ saturation levels >92–94 % at room air, stabilization of clinical presentation, adequate oral intake of fluids and feeds (>75 %), adequacy of the family unit in terms of providing monitoring and possible continuation of therapy at home, possibility, if necessary, of obtaining pediatric health care assistance locally. To date, there is no specific treatment for viral bronchiolitis, and the mainstay of therapy is supportive care, i.e. nasal suctioning and nebulized 3 % hypertonic saline, assisted feeding and hydration, humidified O_2_. The possible role of any pharmacological approach is still debated, and until now there is no evidence to support the use of bronchodilators, corticosteroids, chest physiotherapy, antibiotics or antivirals. Nebulized adrenaline may sometimes be useful in the Emergency Room. Since a specific etiological treatment is lacking, prophylaxis and prevention, especially in children at high risk of severe infection, play a fundamental role. Environmental preventive measures minimize viral transmission in the hospital, in the outpatient setting and at home. Pharmacological prophylaxis with palivizumab for RSV bronchiolitis is indicated in specific categories of children at risk during the epidemic period.

The inter-society consensus document [37] on treatment and prevention of bronchiolitis is an important tool for pediatricians and neonatologists. Considering the paucity of critical care research in pediatric viral bronchiolitis, intensive care practitioners must substantially rely on individualization of therapies based on bedside clinical assessments. However, with the introduction of new diagnostic and respiratory technologies, our ability to support critically ill infants with acute viral bronchiolitis will continue to advance.

Plastic bronchitis (PB) is a rare complication of a variety of respiratory diseases that may secondarily complicate the post-operative course of Fontan surgery in patients with cyanotic congenital heart disease (CHD). Bronchial casts with rubber-like consistency develop acutely and may cause severe life-threatening distress. The pathophysiology of PB remains to be elucidated, and its management is not yet well defined. It has been suggested that high pulmonary venous pressures may lead to an abnormal response from the respiratory epithelium and/or lymphatic dysfunction, thus resulting in excess mucus production and cast formation [38]. An underlying genetic predisposition associated with an inflammatory trigger has also been postulated to explain the abnormal mucin hypersecretion. PB has been classified in type 1 (inflammatory) and type 2 (noninflammatory) according to the characteristics of casts. Colaneri et al. [39] report the case of an adolescent with CHD and primary ciliary dyskinesia (PCD) who developed features of PB and severe respiratory failure 7 years after Fontan operation. The occurrence of PB in this patient permitted to hypothesize that decreased mucociliary clearance was an adjunctive trigger for the development of PB. Seven years after the Fontan operation the patient was admitted to the Intensive Care Unit because of severe respiratory distress, with scattered, harsh breath sounds at chest auscultation. Arterial blood gas analysis showed pH 6.9, PaO_2_ 61 mmHg, SaO_2_ 88 %, PaCO_2_ 150 mmHg, and the patient was immediately mechanically ventilated. Chest radiograph (CR) revealed patchy consolidation in the right middle lobe and mild pleural effusion. A flexible fiberoptic bronchoscopy revealed large, mucinous casts partially obstructing the airway tree, and rigid bronchoscopy was necessary to remove the casts. An immediate improvement in oxygenation was obtained. Histological examination revealed mostly mucous material with fibrin and rare inflammatory cells. Cultures taken from the casts were negative. Mechanical ventilation was withdrawn on hospital day 12. Treatment with nebulized tissue plasminogen activator (t-PA) was added. Over the next few days, the child started expectorating thinner bronchial secretions and his condition improved gradually, with clearing of areas of atelectasis and no recurrence of respiratory symptoms. The patient was discharged receiving t-PA 5 mg for four times daily by nebulisation for 2 months. The patient received instructions to reintroduce a course of t-PA at the re-onset of symptoms or signs (cough, expectoration or chest discomfort). During the 12 month follow-up, this therapeutic regimen was reintroduced eight times with complete relief, no side effects and no further hospital admission underlining that this regimen may be useful in Fontan patients with plastic bronchitis refractory to conventional therapy [40].

Idiopathic pulmonary hemosiderosis (IPH) is a rare disorder responsible for recurrent episodes of diffuse alveolar haemorrhage, characterized by the triad of hemoptysis, iron deficiency anemia and diffuse pulmonary infiltrates. The clinical course is exceedingly variable especially in children, and a substantial proportion of this age group remains undiagnosed. The rarity of this disease and the variable clinical course results in many diagnostic pitfalls especially in children. This is probably due to the fact that iron deficiency anemia may be the first and only manifestation of IPH, preceding other symptoms and signs by several months. Delayed diagnosis of IPH favours complications and the start of treatment at a stage when pulmonary fibrosis has already developed, with a consequently poorer prognosis. Bakalli et al. [41] report the case of a 13 year-old girl who, at admission, appeared very pale, with extreme tiredness, inability to stay upright. Dyspnea and tachypnea with very low oxygen saturation (SaO_2_) in room air (78 %), and low systemic blood pressure (80/40 mmHg) were also present. For several months the girl had been treated for iron deficiency anemia with oral iron salts with no response. The child’s condition deteriorated rapidly with significant respiratory failure. Chest X-ray evidenced bilateral alveolar infiltrates. CT images associated with the presence of hemosiderin-laden macrophages called siderophages (pathognomonic of this disease) in gastric lavage fluid confirmed the diagnosis of IPH. Therapy with corticosteroids was started with a partial and transient response. The girl presented recurrent episodes of dyspnea and severe anemia every 2 to 3 months. Due to prolonged therapy, her aspect became progressively *cushingoid*. Azathioprine, the “second line” immunosuppressant recommended in IPH, was subsequently introduced, and the girl’s clinical condition progressively improved. The triad of iron deficiency anemia, hemoptysis and diffuse infiltrates on chest - X ray characterizes the onset of IPH. The clinical course of the disease is exceedingly variable, especially in children. The age of presentation may be bimodal, with a peak in children younger than 5 years of age and in adolescents. Anemia is hypochromic and microcytic, due to the chronic loss of blood. Failure of iron therapy and exclusion of its common causes, prompted authors to consider other diagnostic possibilities including IPH, even though the latter is a rare cause of anemia. Diagnosis of IPH is based on exclusion of other causes of intrapulmonary haemorrhage and systemic diseases. When children present with unexplained anemia and bilateral lung infiltrations, pulmonary haemorrhage should be suspected [42]. In the absence of systemic disease, findings of hemosiderin-laden macrophages in bronchoscopic lavage or gastric aspirate/sputum along with chronic pulmonary symptoms lead to a diagnosis of IPH. Lung biopsy is the gold standard for diagnosis. HRCT scan is useful for early detection of pulmonary fibrosis. It has been reported that steroid treatment is associated with decreased pulmonary bleeding relapses and pulmonary fibrosis. Immunosuppressive therapies, mainly azathioprine and hydroxychloroquine, are generally prescribed in patients with steroid-refractory disease. Since there is a lack of large patient series and inadequate follow-up in previous studies, the prognosis of IPH remains unclear. However, aggressive treatment is associated with prolonged survival and improved prognosis [43].

There is growing recognition that patient-reported outcomes, such as health-related quality of life (HRQoL), are important indicators of patient benefit in clinical trials [44]. This is particularly true for patients with chronic illnesses such as cystic fibrosis (CF), in which disease management is both challenging and lifelong. Patients with CF are maintaining greater lung function and enjoying longer life expectancy; thus, the need for clinical trial end points in addition to pulmonary function indexes is becoming more critical. It has been demonstrated that CF has a significant impact on patients’ HRQoL [45] At present the primary goal of CF physicians is not only to extend patients’ life but also to improve their quality of life. The multicenter study from Bodnar et al. [46] intended to investigate the relationships between demographic variables, disease severity, pulmonary variables, nutritional status and HRQoL in CF patients, and, finally to assess the level of agreement of HRQoL scores between children/adolescents with CF and their parents. Fifty-nine CF patients from five Hungarian CF centers completed the survey. HRQoL was measured using The Cystic Fibrosis Questionnaire-Revised (CFQ-R). Parents were asked to fill out a questionnaire on their smoking habits, educational level and history of chronic illnesses. Disease severity was assessed using the physician-reported Shwachman-Kulczycki (SK) score system. Spirometry, Body Mass Index (BMI) percentile (pc), hospitalization and Pseudomonas aeruginosa (PA) infection were examined as physiologic parameters of CF, and the impact of these factors on HRQoL was also assessed. As far as the results, passive smoking, parental educational level and chronic diseases of parents did not affect the HRQoL of CF patients. In contrast, hospitalization, PA infection and malnutrition had a significant and negative impact on patients’ HRQoL and clinical severity of the disease. There is growing recognition that patient-reported outcomes, such as HRQoL, are important indicators of patient benefits. This is particularly true for patients with chronic illnesses such as CF, in which disease management is both challenging and lifelong. The evaluation of HRQoL provides additional information for clinicians and is a promising additional end point for clinical trials that may translate into better treatment adherence and improved clinical outcomes.

## Conclusions

This synopsis of advances in pediatrics in 2014 provides indications on standards of care, environmental risk factors, mechanisms, novel tests to facilitate diagnosis and new treatment options. Position statement of scientific societies on the management of bronchiolitis is an important support for improving its management. Malnutrition remains a common underestimated problem that requires urgent actions in developing countries. On the other hand, children in Western countries are exposed to cancer risk factors, and this appears consistent with data from Italy. Important progresses have emerged regarding etiopathogenic mechanisms and possible management and treatment in infantile colic and inflammatory bowel disease. Diagnostic tests are improving, and early studies suggest the diagnostic utility of US in diagnosing CAP. Investigations suggest a need to better educate guardians and improve nutritional management of food allergy. Insights on clinical features and pathogens emphasized the preventive role of varicella vaccine in acute cerebellitis, and of MP in CAP. Recommendations on asphyxia and KMC in neonates have been highlighted. Insights in treatments have evidenced benefits from paracetamol in PDA. Advances in our understanding of less common diseases such as cystic fibrosis, plastic bronchitis, and IPH facilitate diagnosis and management.

## Abbreviations

IBD: Inflammatory bowel disease
CR: Chest radiography
US: Ultrasonography
CAP: Community-acquired pneumonia
MP: *Mycoplasma pneumoniae*
PDA: Patent ductus arteriosus
COX: Cyclo-oxygenase
KMC: Kangaroo mother care
NHL: Non-Hodgkin Lymphoma
PB: Plastic bronchitis
CHD: Congenital heart disease
t-PA: Tissue plasminogen activator
IPH: Idiopathic pulmonary hemosiderosis
CF: Cystic fibrosis
HRQoL: Health-related quality of life
